# Integument colouration and circulating carotenoids in relation to urbanisation in Eurasian kestrels (*Falco tinnunculus*)

**DOI:** 10.1007/s00114-023-01874-5

**Published:** 2023-09-22

**Authors:** Petra Sumasgutner, Tom Nilles, Alba Hykollari, Manuela Merling de Chapa, Caroline Isaksson, Lukas Hochleitner, Swen Renner, Leonida Fusani

**Affiliations:** 1 Konrad Lorenz Research Center, Core Facility for Behavior and Cognition, University of Vienna, Grünau/Almtal, Vienna, Austria; 2 Department of Behavioral and Cognitive Biology, University of Vienna, Vienna, Austria; 3 Natural History Museum Vienna, Vienna, Austria; 4 Research Institute of Wildlife Ecology, University of Veterinary Medicine Vienna, Vienna, Austria; 5 Leibniz Institute for Zoo and Wildlife Research, Berlin, Germany; 6 Hessian Agency for Nature, Environment and Geology, Biodiversity Center, Giessen, Germany; 7 Department of Biology, Lund University, Lund, Sweden; 8 Konrad Lorenz Institute of Ethology, University of Veterinary Medicine, Vienna, Austria

**Keywords:** Urbanisation, Dietary antioxidants, Colour ornaments, Circulating carotenoids, Lutein, Zeaxanthin, Eco-physiology, Environmental quality, Urban stressors, Raptors

## Abstract

Urbanisation is one of the biggest environmental challenges of our time, yet we still lack an integrative understanding of how cities affect behaviour, physiology and parasite susceptibility of free-living organisms. In this study, we focus on carotenoids, strictly dietary micronutrients that can either be used as yellow-red pigments, for integument colouration (signalling function), or as antioxidants, to strengthen the immune system (physiological function) in an urban predator, the Eurasian kestrel (*Falco tinnunculus*). Kestrels are specialised vole hunters but shift to avian prey in cities where diurnal rodents are not sufficiently available. This different foraging strategy might determine the quantity of carotenoids available. We measured integument colouration, circulating carotenoids in the blood and ectoparasite burden in kestrels along an urban gradient. Our results showed that nestlings that were raised in more urbanised areas displayed, unrelated to their ectoparasite burden, a paler integument colouration. Paler colours were furthermore associated with a lower concentration of circulating carotenoids. These findings support the hypothesis that the entire urban food web is carotenoid deprived and only prey of low quality with low carotenoid content is available (e.g. fewer carotenoids in urban trees, insects, small birds and finally kestrels). The alternative hypothesis that nestlings allocate carotenoids to reduce physiological stress and/or to cope with parasites rather than invest into colouration could not be supported. Our study adds to existing evidence that urban stressors negatively affect carotenoid production in urban areas, a deficiency that dissipate into higher trophic levels.

## Introduction

Urbanisation has greatly altered the environment to serve the need of one species: humans Seto, Güneralp et al. (2012). The process of urbanisation transforms natural areas to artificial environments characterised by high levels of night light, noise, and air pollution, changes in resource availability and quality (Sumasgutner, Cunningham et al. 2023), competition with alien species and alteration of species composition which includes pets, livestock and high densities of humans (Faeth et al. 2005; Shanahan et al. 2014). These new challenges presented by cities cause a huge negative impact in reducing the local biodiversity, where only a few species persist (so-called biotic homogenisation; McKinney 2006). Typical behavioural adjustments seen in urban environments (Lowry, Lill et al. 2013) include the usage of anthropogenic food (Meyrier, Jenni et al. 2017; Stofberg, Cunningham et al. 2019), nesting structures or nesting material (Reynolds, Ibáñez-Álamo et al. 2019). The success of an animal in urban areas could therefore depend on its behavioural flexibility (or phenotypic plasticity) that might include personality traits like boldness or aggressiveness (Lowry, Lill et al. 2013).

Urban exploiter species reach higher densities in cities than in a surrounding non-urban habitat (Møller, Diaz et al. 2012). These higher densities are usually explained by the high availability of attractive artificial nesting sites, especially for cavity nesters (Sumasgutner, Schulze et al. 2014a; Meyrier, Jenni et al. 2017). However, limited accessibility of high-quality food resources in cities could have negative health or fitness consequences (Sumasgutner, Nemeth et al. 2014b; Meyrier, Jenni et al. 2017; Catto, Sumasgutner et al. 2021). Thus, urban areas do not necessarily constitute optimal habitat for urban exploiters, as they can mislead animals by, for example, a high number of nesting opportunities into areas with low food availability and quality.

Raptors have successfully colonised cities across the globe (Boal and Dykstra 2018; Kettel, Gentle et al. 2018; McPherson, Sumasgutner et al. 2021; Headland, Colombelli-Négrel et al. 2023). Whether a raptor species is more or less urban tolerant largely depends on the body size, with smaller raptors being more abundant in cities (Cooper, Shultz et al. 2022; Headland, Colombelli-Négrel et al. 2023), and on the prey type, with avian specialists showing higher breeding success in cities (Kettel, Gentle et al. 2018). For example, peregrine falcons (*Falco peregrinus*), which are specialised in hunting birds, show larger clutch and brood sizes (Kettel, Gentle et al. 2019; Sumasgutner, Jenkins et al. 2020), while Eurasian kestrels as typical rodent hunters suffer from lower breeding success (Sumasgutner, Nemeth et al. 2014a) in urban centres. These findings highlight that diet has an important role to play in the success of urban-living raptors.

Every prey type has characteristic nutritional components and differentiates in caloric content and micronutrient composition (Fargallo, Navarro-Lopez et al. 2020). While voles are rich in calories, they are restricted in their carotenoid content (Goodwin 1980). In contrast, insectivores like some birds, shrews and lizards, and insects themselves, have an overall higher carotenoid content from the plant they feed on (Goodwin 1980) but might be more energy-demanding to catch and/or provide less calories. Carotenoids are micronutrients that are strictly dietary in vertebrates and can thus constitute a limited resource (Hill and McGraw 2006). Health-related functions of carotenoids include antioxidant capacities to limit oxidative damage triggered by unfavourable environmental conditions such as pollution or to deal with parasite infections as immune-stimulant (von Schantz, Bensch et al. 1999; Martinez-Padilla, Mougeot et al. 2007; Pérez-Rodríguez, Martínez-Padilla et al. 2013). Correlations between carotenoids and parasite infections have been demonstrated in several avian systems, including passerines (Pap, Vágási et al. 2011; Figuerola, López et al. 2014), grouse (Martinez-Padilla, Mougeot et al. 2007) and raptors (De Neve, Fargallo et al. 2008). The different signalling and physiological functions of carotenoids can thus result in a trade-off whereby intense yellow-red colouration is known to be important for mate selection, signalling a strong, healthy individual (Weaver, Santos et al. 2018).

The carotenoid content of prey determines the carotenoid availability for the raptor, which can then be allocated either as yellow-red pigments for integument colouration or as antioxidants to health-related functions (Casagrande, Costantini et al. 2009). Within this physiological trade-off, both components of the carotenoid pathway can be measured, as carotenoid-based colouration of skin or feathers or as circulating carotenoids in the bloodstream.

The Eurasian kestrel population in Vienna, Austria, is one of the densest breeding populations of raptors in any city worldwide (Sumasgutner, Nemeth et al. 2014a; Sumasgutner, Schulze et al. 2014b. However, high population density does not necessarily indicate a viable population: high nestling mortality with low overall productivity (Sumasgutner, Nemeth et al. 2014a; Sumasgutner, Schulze et al. 2014b) and impaired physiological health (Sumasgutner, Adrion et al. 2018; Wemer, Hegemann et al. 2021) are characteristics of this study system that cannot be explained by density dependence alone (Sumasgutner, Nemeth et al. 2014a). Urban kestrels display a more generalists’ diet (Sumasgutner, Krenn et al. 2013) and include more nocturnal rodents (house mice and *Apodemus* species), birds, reptiles and insects compared to their vole specialist conspecifics in suburban and rural areas. The high proportion of alternative prey in the city centre (Sumasgutner, Krenn et al. 2013) and the higher breeding success in proximity to green spaces, even within urban areas (Sumasgutner, Schulze et al. 2014a), both suggest that kestrels are likely to hunt near their nests. Indeed, first tracking studies in Rome, Italy, confirm them to be city-bound with rather small home range sizes (Costantini and Dell’Omo 2020). These dietary shifts and/or decreased foraging efficiencies in catching alternative prey result in an overall lower amount of food for nestlings. Furthermore, the constant exposure to stressors in urban areas (i.e. light, noise and air pollution or urban heat; see review in Sumasgutner, Cunningham et al. (2023)) might impact health and fitness.

Previously, we have shown that urban nestlings show paler integument colouration (Sumasgutner et al. 2018), but thus far, we could not disentangle if this is due to a lower carotenoid content in urban prey or due to a higher demand for carotenoids as dietary antioxidants in the immune system. We identified circulating carotenoids in the bloodstream as an important missing link to draw a more complete picture on the carotenoid trade-off in the city. Our findings on paler colouration in urban kestrels were already surprising, as the known change of main prey types from voles to birds, lizards and insects in urban kestrels would predict a larger intake of carotenoids in the city, which could then be visible in a more intense colouration (i.e. the trophic chain would start with a high carotenoid availability through birds, lizards and insects (García-Heras, Arroyo et al. 2017)). However, carotenoid content of primary producers can in turn be reduced due to pollution in urban areas in a way that plants synthesise less carotenoids when exposed to environmental stress. This environmental paucity of carotenoids could be an alternative explanation for the found paler colouration in kestrels (Sumasgutner, Adrion et al. 2018) and other urban-dwelling species (Sillanpaa, Salminen et al. 2008; Jensen, Ziegler et al. 2022).

Given this background, we explore two possible explanations for paler carotenoid-based colouration found in urban kestrels. First, if the urban environment is overall lower on carotenoids, we would expect both integument colouration and the level of circulating carotenoids in urban kestrels to be lower than in suburban and rural kestrels. Second, if carotenoids are preferentially allocated towards maintaining a high circulating level to defeat reactive oxygen species as a consequence of exposure to urban stressors and/or ectoparasite infection, we would expect paler colouration but elevated levels of circulating carotenoids in the blood together with higher ectoparasite burdens.

## Material and methods

### Study system

**Fig. 1:**
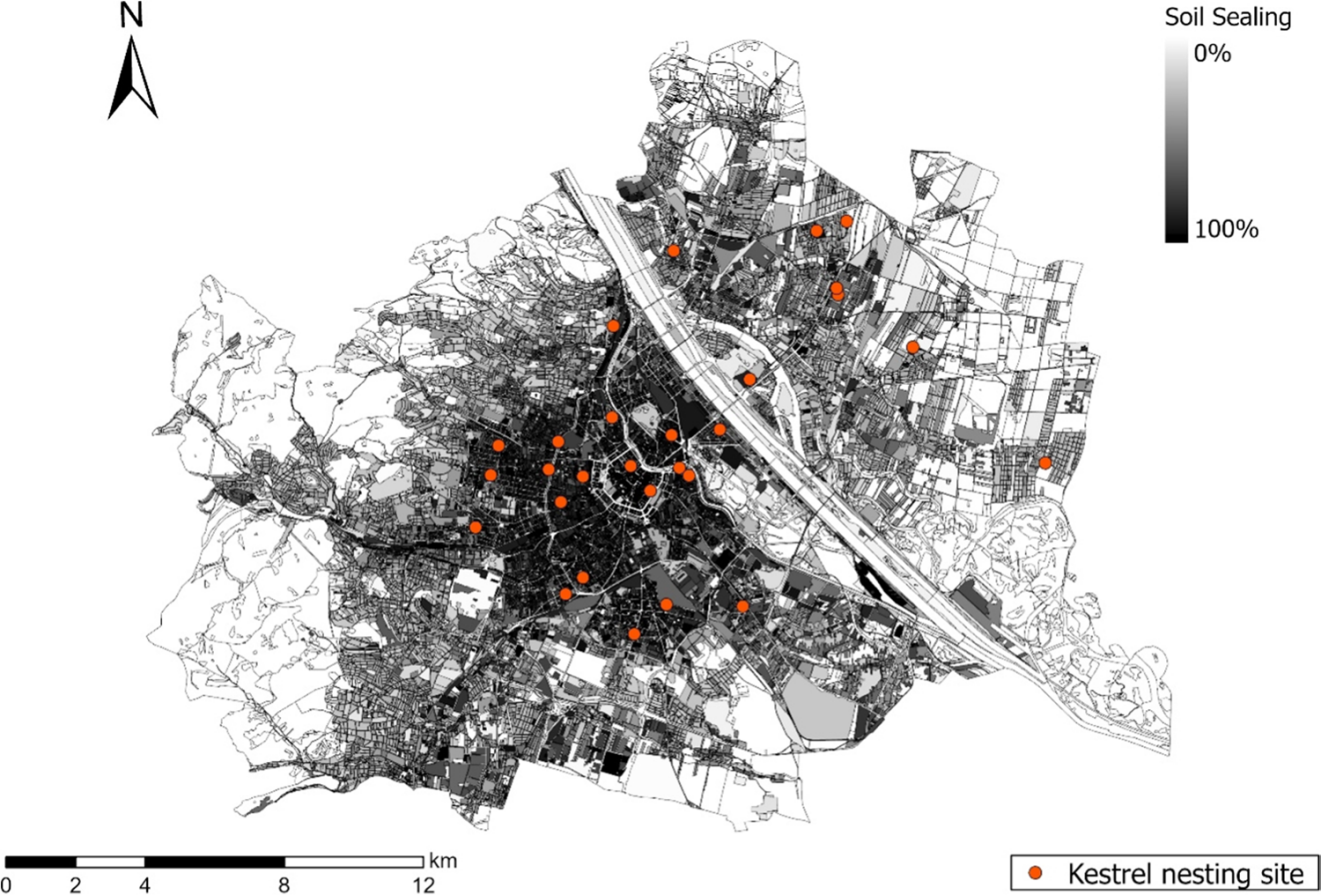
Urban study area in Vienna, Austria (243 km^2^). The urban gradient was quantified by the percentage of sealed soil and displayed from light grey to black (white areas (< 1%) are mainly agriculture areas in the east and large forested areas in the west, which were not systematically monitored). The locations of kestrel nesting sites sampled for this study (*n* = 28) are represented in orange

**Fig. 2:**
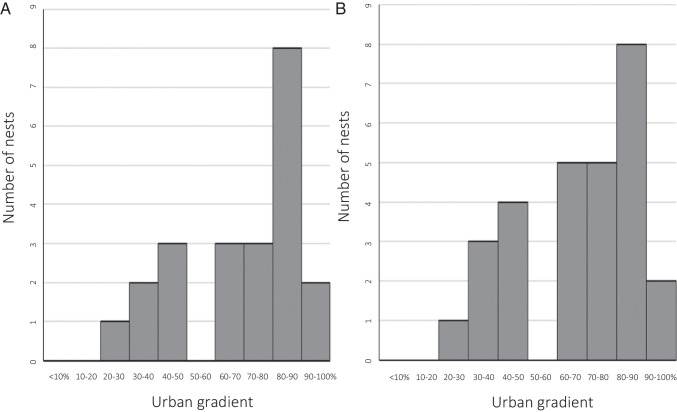
Frequency of individual kestrel nesting sites where **A** circulating carotenoids were quantified (*n* = 22) and where **B** integument colourations were measured along the urban gradient in 2020 and 2021

The study area is the city of Vienna, Austria (48° 12′N, 16° 22′E; 415 km^2^; 150 ± 500 m asl; 1.9 million inhabitants), where the density of kestrels is estimated to be 89–122 breeding pairs per 100 km^2^ in urbanised areas (Sumasgutner, Nemeth et al. 2014a). We quantify the degree of urbanisation GIS-based as percentage of sealed surfaces within a buffer circle of *r* = 500 m (78.5 ha) around each nest (Fig. 1; see details in the method in Sumasgutner, Nemeth et al. (2014b)), corresponding to kestrel home range sizes in urban areas (Beichle 1980; Costantini and Dell’Omo 2020). The resulting continuous measurement of urbanisation (Seress, Lipovits et al. 2014) quantifies variation in several urban factors simultaneously, i.e. infrastructure (physical measure of sealed surface area), human population (disturbance) and the exposure to anthropogenic stressors including artificial light at night (see Stathakis et al. 2015; Zhang & Seto 2013 for validation studies). The nesting sites were sampled for carotenoid-based integument colouration and for plasma volumes of circulating carotenoids in 2020 (*n* = 19) and 2021 (*n* = 17) and were located in areas between 25 and 95% sealed surface area. Because some nest sites were sampled in both years, the effective sample size is 28 nests for the analysis on integument colouration and 22 nests for the analysis on circulating carotenoids (Fig. 2). Year was included as covariate in both models to account for a potential annual variation.

### Field procedure

Each kestrel was banded with a uniquely coded metal ring (provided by the Austrian Ornithological Centre; “Österreichische Vogelwarte”) and an alpha-alpha–coded colour ring between 10 and 25 days after hatching. Kestrel nestlings hatch asynchronous, why large age discrepancies can exist within one brood, explaining the variation in sampling age. In addition, the birds were measured and weighed (according to the Standard Manual of the Austrian Ornithological Centre), and the number of ectoparasites (species *Carnus hemapterus*) per individual was counted without removing them from the body. The age of the nestlings was determined based on their wing length according to Kostrzewa and Kostrzewa (1987). Importantly, age was not confounded with the urban gradient (i.e. the sampling age was not earlier or later in more or less urbanised areas; Pearson correlation coefficient *r* = −0.024), nor was it confounded with the number of ectoparasites (i.e. younger nestlings tend to have more ectoparasites (Roulin, Gasparini et al. 2008), *r* = −0.23). To account for the hatching asynchrony, each nestling was assigned a hatch rank, as senior (“1”, first hatched/largest), junior (“3”, last-hatched/smallest) or middle (“2”, all the nestlings in between) sibling (see Martínez-Padilla and Viñuela 2011 for more details). To derive a nestlings’ body mass index, we extracted the residuals of a linear model with body mass (in g) as the response and wing length (in mm, linear and quadratic) and sex (males being 20% smaller than females in body mass, Village 1990) as explanatory variables, following Roulin et al. (2007).

### Carotenoid-based integument colouration

The colour of the skin (cere, orbital ring and tarsus) was evaluated by comparing the bare parts of the nestlings with a reference colour chart (Michel 2000) and recorded with digital photographs for further extraction of spectral measurements using GIMP 2.10.28. We selected a homogeneous area at the photographs to obtain hue (H), saturation (S) and brightness (B) for each bare part and the corresponding colour chart. The hue represents the perceived colour (e.g. yellow, orange, red) using values ranging from 0° to 360°, the saturation indicates the intensity and purity (in %) of the colouration, and brightness indicates the “quantity of white” (in %) in the colour (see García-Heras et al. 2017 for further details; and illustration in Appendix Figure 8). The colour measurements were taken from 112 nestling across 28 different nest sites over the 2-year period. Due to the high similarity between the colour of the cere and the orbital ring, we averaged the values into one hue, saturation and brightness value, respectively, as “face colouration”. We concluded this “high similarity” by the fact that face colouration could always be matched with the same colour chart reference, while tarsus colouration usually required a different colour field on the chart as reference. Face colouration was furthermore positively correlated with tarsus colouration measurements (hue: *r* = 0.04, *p* < 0.0001; saturation: *r* = 0.56, *p* < 0.0001; brightness: *r* = 0.41, *p* < 0.0001). To reduce the number of variables, a principal component analysis (PCA) was run on the 6 remaining colouration variables (i.e. hue, saturation, brightness of face and tarsus, respectively). The first principal component (PC1-colouration) explained 82.3% of the variance, while the second and third principal components (PC2-colouration and PC3-colouration) explained further 7.2% and 5.8%, respectively (Appendix Table 3). PC1-colouration represents face hue (e.g. bluish or yellowish), where high axis values consist of low face hue values (yellowish face colouration) and low axis values of high face hue values (bluish face colouration) (Appendix Figure 9). The PC2-colouration represents saturation (i.e. intensity) of colouration, which is usually more strongly pronounced in the tarsus than in the face. High axis values of PC2-colouration indicate a lower saturation. The PC3-colouration represents brightness (i.e. whiteness) of colouration, with high axis values indicating lower brightness.

### Plasma volumes of circulating carotenoids

Blood samples were collected after disinfecting the skin with an alcohol swab, by puncturing the brachial vein with a sterilised 21-gauge needle and extracting the blood with a 220-μL capillary tube. The blood was stored in a heparinised Eppendorf vial and kept on ice in a thermos until the sample was centrifuged for 15 min at 3000 rpm to separate the plasma from the red blood cells within 4 h after sampling. The plasma was stored at −60 °C until further processing.

After thawing the blood samples for ~30 min on ice, 20-μl plasma was transferred into an Eppendorf tube and mixed with 200-μl high-performance liquid chromatography (HPLC) grade acetone and the internal standard (IS) (600-μM retinyl acetate Sigma + 1-mM tocopheryl acetate Sigma). The tube was vortexed for 10 sec, a 100-μl tert-butyl methyl ether was added, and then vortexed again for another 10 sec. The samples were centrifuged at 10 °C for 5 min at 13,000 rpm. The supernatant was evaporated with a rotary evaporator (Rotavapor) under vacuum for 30 min. The dried sample was dissolved in a 100 μl of the HPLC mobile phase (methanol-acetonitrile (30:70 v/v)) and transferred to a HPLC glass vial and injected into the HPLC system. The HPLC method was carried out with a Thermo Scientific UltiMate 3000 adapted to a Develosil RPAqueous RP-30 HPLC column (250 × 4.6 mm I.D.; Nomura Chemical Co., Ltd., Japan). An isocratic system (HP 1050 Series Isocratic Pump) was applied at a constant flow rate of 1.2 mL/min for 26 min. Carotenoids were detected at 450 nm using an Ultimate 3000 Diode Array Detector UV/VIS detector, whereas the internal standards were detected at 210 and 325 nm. Lutein and zeaxanthin were identified as the two main carotenoids. The data analysis was carried out with the Thermo Scientific™ Chromeleon™ chromatography data system (CDS) software. The concentration of lutein and zeaxanthin was expressed in nmol/mL and summed to a total “circulating carotenoid” concentration for 88 kestrel nestlings across 22 different nest sites over the 2-year period. We did not consider zeaxanthin any further, due to overall low values, following recommended methods from other raptor species (García-Heras, Arroyo et al. 2017).

### Statistical analyses

All statistical analyses were carried out in R version 4.0.1 (R Development Core Team 2020) using the “lme4” (Bates, Maechler et al. 2015) package, and results were visualised using the “effects” (Fox 2003) and “ggplot2” (Wickham 2009) packages. Residual distributions of the models were inspected visually to assess model fit by evaluating the model criticism plots produced by the “plot” function in the base package and the “mcp_fnc” function in the “LMERConvenienceFunctions” package (Tremblay and Ransijn 2015). The significance of the fixed effects was calculated with the “Anova” function type III in the “car” package (Fox and Weisberg 2019) and was accepted at *p*≤0.05, and the confidence intervals were set to 95%. Nest site (ID of nest location) was considered as a random factor in each model to account for pseudoreplication arising from the lack of independence of nestlings within one brood and considering the same nest site in consecutive years (2020 and 2021), potentially used by the same breeding pair. Multiple covariates were added to control for their potential influence on carotenoid trade-off: urban gradient (% cover of sealed surface area) and number of ectoparasites as main predictors as per our hypotheses, age (in days), hatch rank (factor in three levels: junior, middle, senior), body mass index, brood size and study year. All quantitative variables (i.e. concentration of circulating carotenoids (log transformed to archive normality), urban gradient, number of ectoparasites, age, brood size) were scaled to ensure that the effect sizes were on a comparable scale.

First, to determine the relationship between the different colour variables (i.e. face hue: PC1-colouration, saturation: PC2-colouration, and brightness: PC3-colouration) and the circulating carotenoids, a generalised linear mixed model (GLMM) per colour variable was created in which the colour variable was the response variable and the concentration of the circulating carotenoids together with study year were the explanatory variables.

Second, to test the influences of different variables (urban gradient, number of ectoparasites, age, hatch rank, body mass index, brood size and study year) on the colour variables, another set of GLMMs (one per colour variable) was created in which integument colouration was the response variable.

Third, to investigate the effects of the different variables (urban gradient, number of ectoparasites, age, hatch rank, body mass index, brood size and study year) on the concentration of circulating carotenoids, a last GLMM was created.

## Results

### Carotenoid-based colouration and circulating carotenoids

**Fig. 3:**
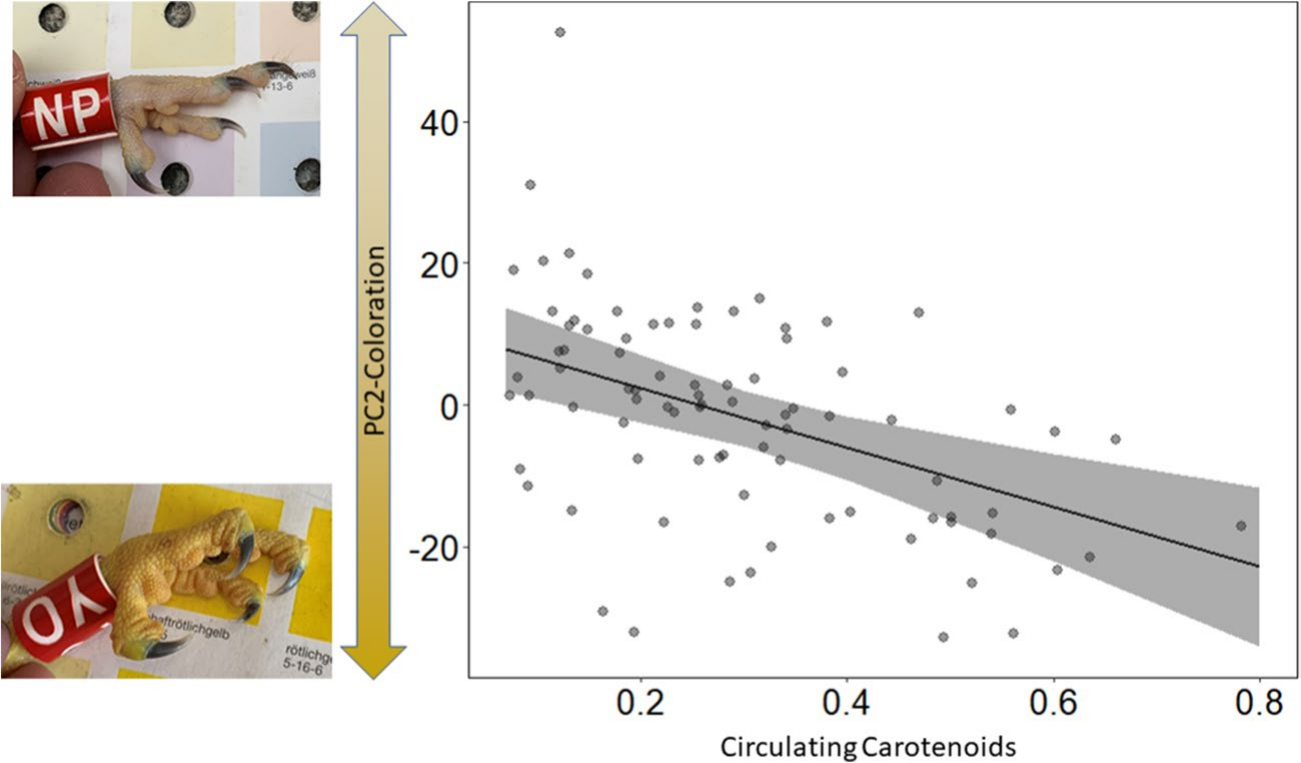
Relationship between saturation of the colouration (PC2-colouration) and the circulating carotenoids concentration (in nmol/mL, log transformed) in kestrel nestlings

The saturation of the colouration (PC2-colouration) was negatively correlated with the concentration of circulating carotenoids in nestlings (*χ*^2^ = 16.442, *p* < 0.0001) indicating that higher volumes of circulating carotenoids were associated with a more saturated (purer) colouration of integuments (Fig. 3) and vice versa. The higher the concentration of lutein and zeaxanthin in the plasma, the more yellow the skin appears. There was no obvious association between circulating carotenoids and face hue (PC1-colouration; *χ*^2^ = 0.577, *p* = 0.447) or brightness (PC3-colouration; *χ*^2^ = 0.283, *p* = 0.595).

### Association between colouration, urbanisation and ectoparasites

**Fig. 4:**
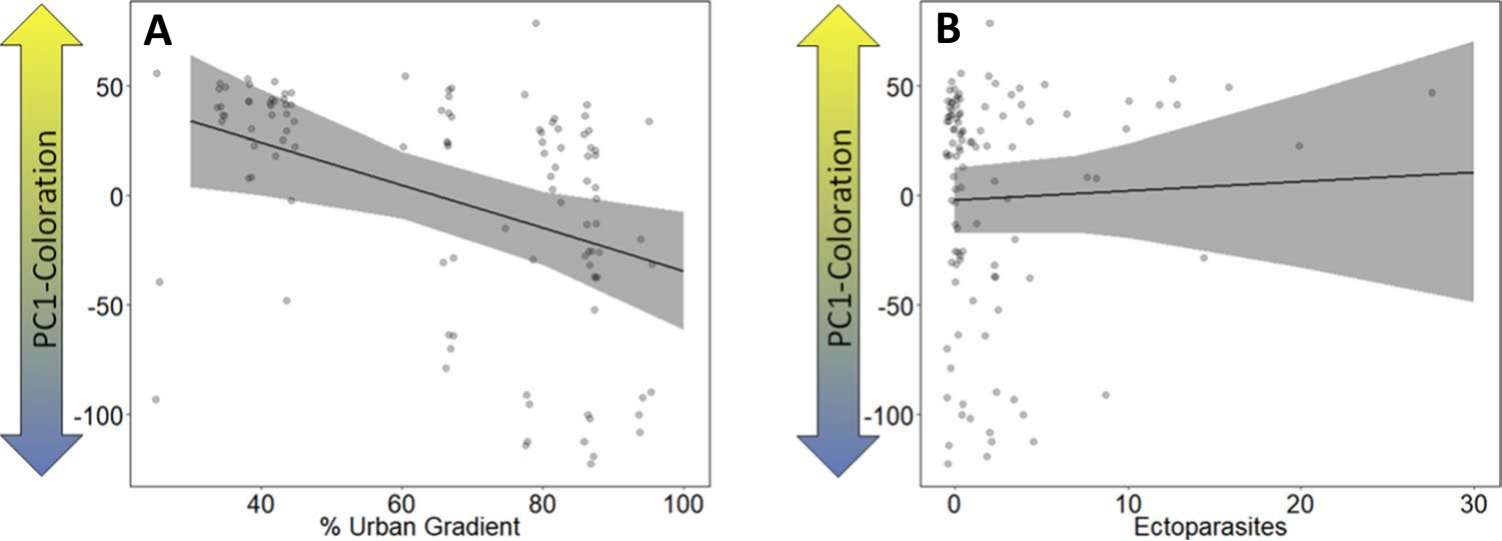
Results of the model testing for the relation between the face hue (PC1-colouration) and **A** the urban gradient (in %) (*p* = 0.006) and **B** number of ectoparasites (*p* = 0.680). Plots show raw data in background scatter, effect sizes of GLMM and 95% confidence intervals. Model outputs are provided in Table 1

PC1-colouration decreased significantly with an increasing urban gradient, which means that face hue became more bluish and less yellowish towards the city centre (*χ*^2^ = 7.705, *p* = 0.006; Table 1; Fig. 4A). No relationship was found between face hue (PC1-colouration) and the number of ectoparasites (*χ*^2^ = 0.171, *p* = 0.680; Table 1; Fig. 4B).

**Table 1:** Results of the GLMM testing the effects of urban gradient, age, brood size, hatch rank, ectoparasites, body mass index and year as explanatory variables on the colouration of the kestrel nestlings (face hue (PC1-colouration), saturation (PC2-colouration), brightness (PC3-colouration); see also Appendix Table 3) as response variable

Parameters	Estimate	SE	Chi-square	Df	*p*-value
Face hue (PC1)					
Intercept	−5.102	49.242	0.011	1	0.917
Urban gradient	−0.980	0.353	7.705	1	**0.006**
Age	2.491	1.814	1.886	1	0.680
Brood size	8.431	6.605	1.629	1	0.170
Hatch rank^1^			4.152	2	4.152
Hank rank (middle)	−7.956	9.430			
Hatch rank (senior)	12.673	11.063			
Ectoparasites	0.432	1.045	0.171	1	0.680
Body mass index	0.289	0.227	1.629	1	0.203
Year^2^			0.070	1	0.791
Year (2021)	−2.597	9.793			
Saturation (PC2)					
Intercept	13.772	14.238	0.94	1	0.333
Urban gradient	0.249	0.107	5.39	1	**0.020**
Age	−1.217	0.523	5.42	1	**0.019**
Brood size	−3.388	1.906	3.16	1	0.076
Hatch rank^1^			3.80	2	0.150
Hatch rank (middle)	−1.929	2.578			
Hatch rank (senior)	3.498	3.058			
Ectoparasites	0.304	0.289	0.292	1	0.292
Body mass index	0.028	0.064	0.192	1	0.661
Year^2^			0.190	1	0.663
Year (2021)	−1.197	2.746			
Brightness (PC3)					
Intercept	43.043	12.406	12.036	1	**< 0.001**
Urban gradient	−0.156	0.080	3.824	1	0.051
Age	−1.585	0.458	11.964	1	**< 0.001**
Brood size	−0.359	1.668	0.046	1	0.830
Hatch rank^1^			2.773	2	0.250
Hank rank (middle)	−1.200	2.709			
Hatch rank (senior)	−4.976	3.101			
Ectoparasites	−0.238	0.289	0.679	1	0.410
Body mass index	−0.033	0.060	0.301	1	0.583
Year^2^			9.901	1	**0.002**
Year (2021)	−8.287	2.634			

^1^Hatch rank (junior) was the reference category

^2^Year (2020) was the reference category

*N* = 108 individuals in 28 nests

*Df* degrees of freedom

Significant variables are highlighted in bold

**Fig. 5:**
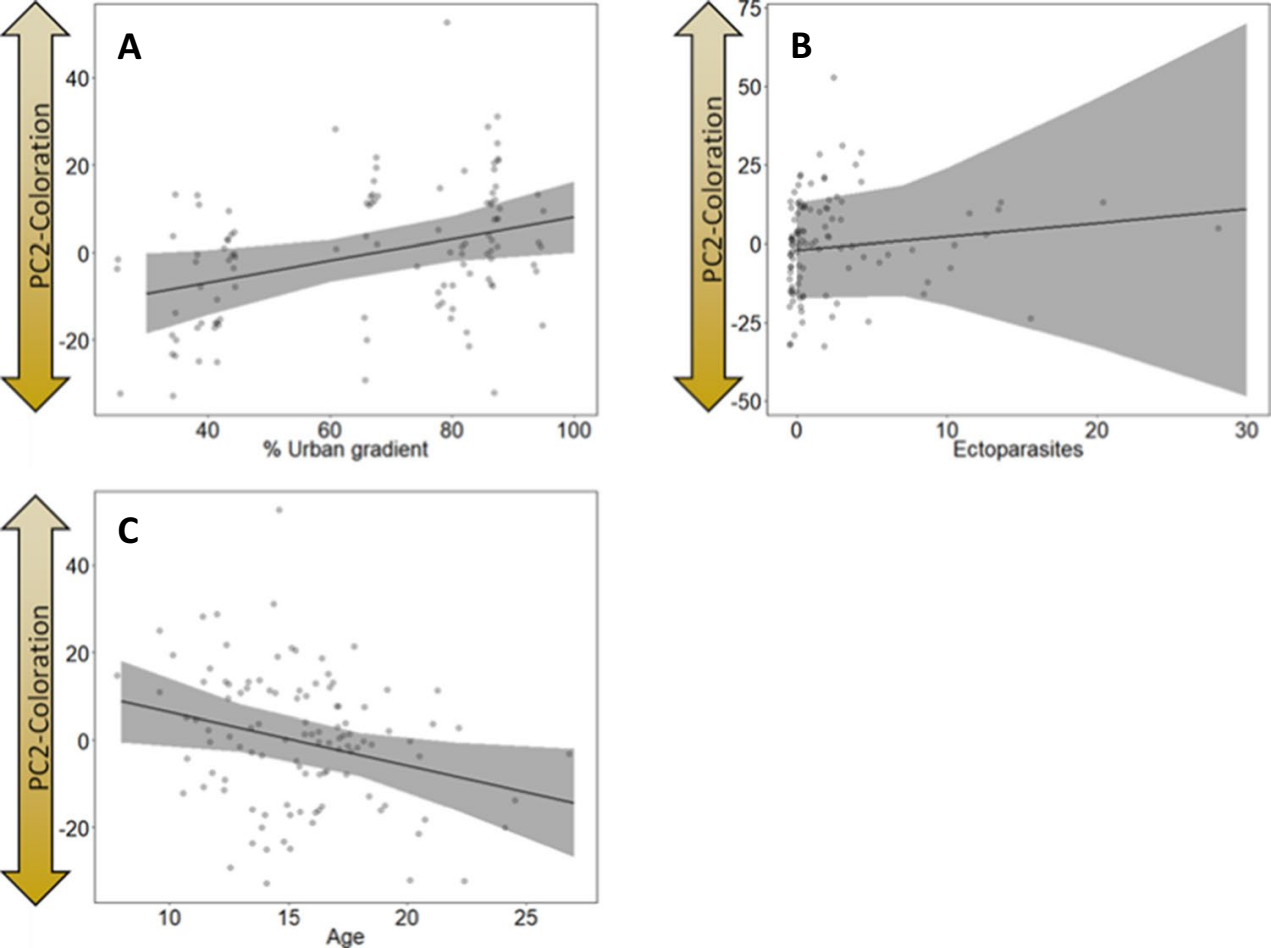
Results of the model testing for the relation between the saturation (PC2-colouration) and **A** the urban gradient (in %) (*p* = 0.02), **B** number of ectoparasites (*p* = 0.292) and **C** the age (in days) (*p* = 0.019). Plots show raw data in background scatter, effect sizes of GLMM and 95% confidence intervals. Model outputs are provided in Table 1

PC2-colouration increased significantly with an increasing urban gradient, which means that saturation of the colouration (relative to white) of the chicks became weaker towards the city centre (*χ*^2^ = 5.39, *p* = 0.020; Table 1; Fig. 5A). No relationship was found between saturation (PC2-colouration) and the number of ectoparasites (*χ*^2^ = 0.292, *p* = 0.292; Table 1; Fig. 5B).

**Fig. 6:**
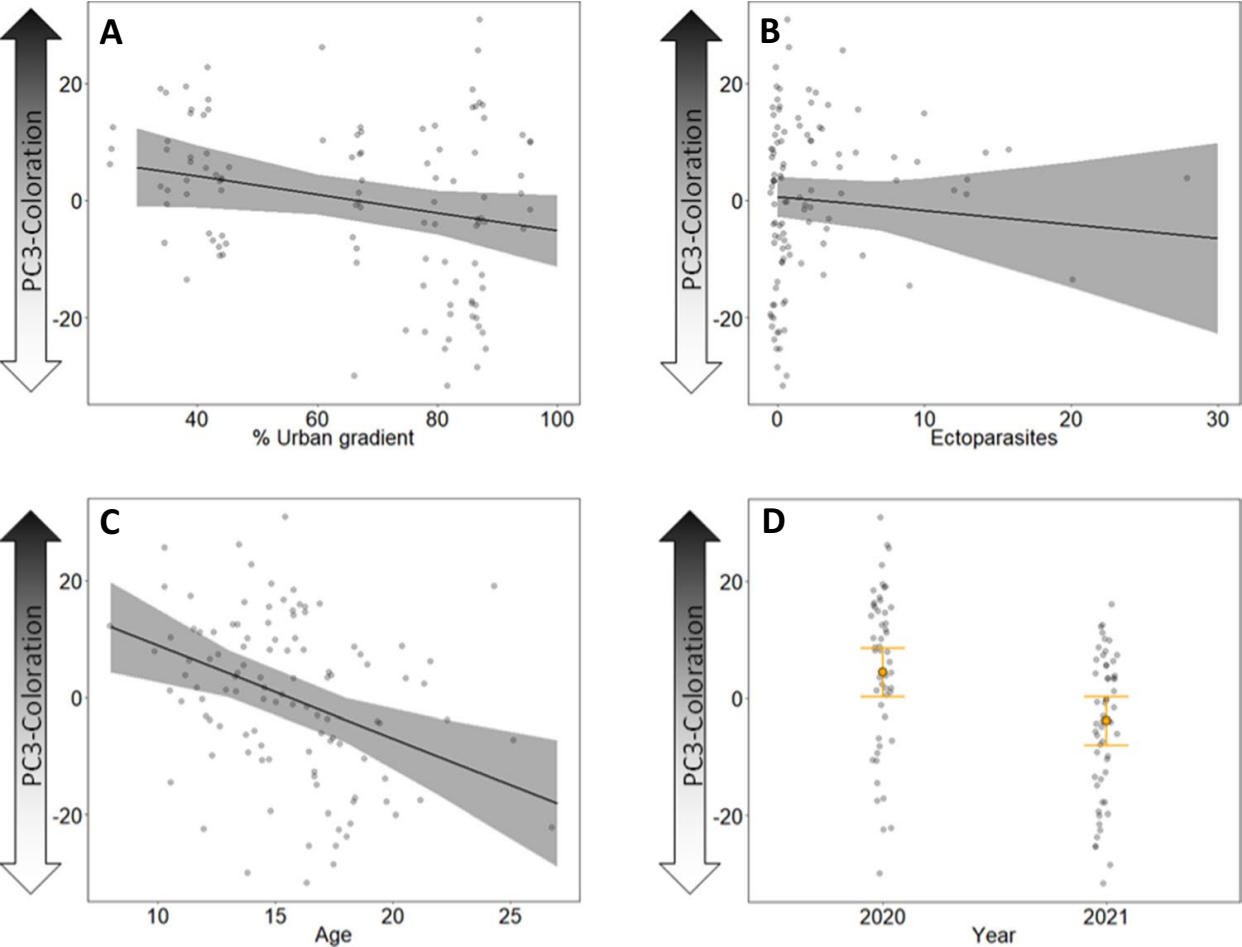
Results of the model testing for the relation between the brightness (PC3-colouration) and **A** the urban gradient (in %) (*p* = 0.051), **B** number of ectoparasites (*p* = 0.410), **C** the age (in days) (*p* < 0.001) and **D** year (*p* = 0.002). Plots show raw data in background scatter, effect sizes of GLMM and 95% confidence intervals. Model outputs are provided in Table 1

PC3-colouration decreased almost significantly with an increasing urban gradient, which would mean that brightness of colouration increased towards the city centre (*χ*^2^ = 3.824, *p* = 0.051; Table 1; Fig. 6A). No relationship was found between brightness (PC3-colouration) and the number of ectoparasites (*χ*^2^ = 0.679, *p* = 0.410; Table 1; Fig. 6B). Furthermore, both PC2-colouration and PC3-colouration decreased significantly with an increasing age, whereby older chicks had a more saturated (more intense) colouration than younger ones (*χ*^2^ = 5.42, *p* = 0.019; Table 1; Fig. 5C) and older chicks were also brighter (*χ*^2^ = 11.964, *p* < 0.001; Table 1; Fig. 6C). We found a significant year effect on PC3-colouration, showing that nestlings in 2021 were brighter than nestlings in 2020 (*χ*^2^ = 9.901, *p* = 0.002; Table 1; Fig. 6D).

### Association between circulating carotenoids, urbanisation and ectoparasites

**Fig. 7:**
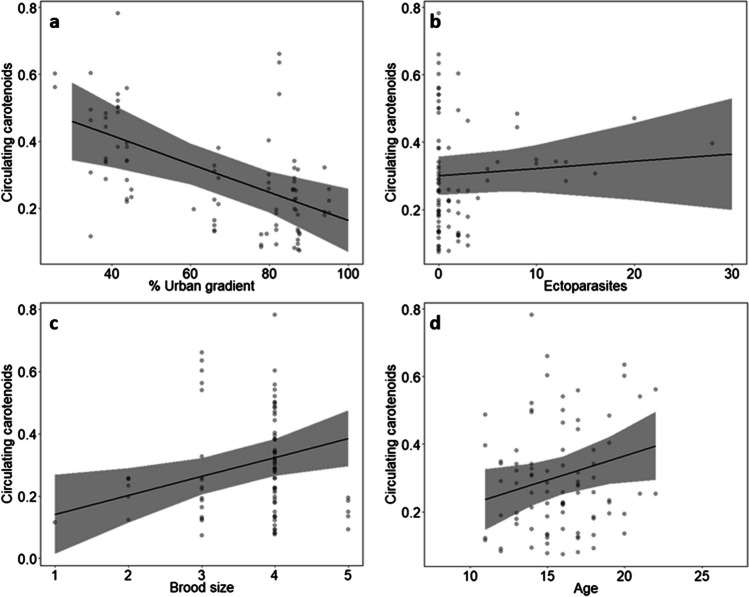
Results of the models testing for the relation between circulation carotenoids concentration (in nmol/mL) and **a** urban gradient (in %) (*p* = 0.001), **b** numbers of ectoparasites (*p* = 0.449), **c** brood size (*p* = 0.009) and **d** age (in days) (*p* = 0.042). Plots show raw data in background scatter, effect sizes of GLMM and 95% confidence intervals. Models outputs are provided in Table 2

The concentration of circulating carotenoids (sum of lutein and zeaxanthin) decreased significantly with an increasing urban gradient, meaning that chicks in more urbanised areas had lower circulating levels of carotenoids compared to those in more suburban areas (*χ*^2^ = 10.714, *p* = 0.001; Table 2; Fig. 7A). There was no obvious association between circulating carotenoids and the number of ectoparasites (*χ*^2^ = 0.574, *p* = 0.449; Table 2; Fig. 7B), but circulating carotenoids were significantly higher in larger broods (*χ*^2^ = 6.831, *p* = 0.009; Table 2; Fig. 7C) and increased significantly with increasing age, in a way that older siblings had higher blood volumes of circulating carotenoids than younger siblings (*χ*^2^ = 4.144, *p* = 0.042; Table 2; Fig. 7D).

**Table 2:** Results of the GLMM testing the effects of urban gradient, age, brood size, hatch rank, ectoparasites, body mass index and year as response variables on circulating carotenoids concentration as explanatory variable in kestrel nestlings

Parameters	Estimate	SE	Chi-square	Df	*p*-value
Circulating carotenoids					
Intercept	0.107	0.167	0.404	1	0.525
Urban gradient	**−0.004**	**0.001**	**10.714**	**1**	**0.001**
Ectoparasites	0.002	0.003	0.574	1	0.449
Age	**0.014**	**0.007**	**4.144**	**1**	**0.042**
Hatch rank^1^			0.820	2	0.663
Hank rank (middle)	−0.001	0.024			
Hatch rank (senior)	0.024	0.033			
Brood size	**0.061**	**0.023**	**6.831**	**1**	**0.009**
Body mass index	-0.0002	0.0006	0.065	1	0.799
Year^2^			1.089	1	0.297
Year (2021)	0.030	0.028			

^1^Hatch rank (junior sibling) was the reference category

^2^Year (2020) was the reference category

*N* = 86 individuals in 22 nests

*Df* degrees of freedom

Significant variables are highlighted in bold

## Discussion

Eurasian kestrels are specialised vole hunters but shift to avian prey in urban environments where diurnal rodents are not sufficiently available (Sumasgutner, Schulze et al. 2014a). This different foraging strategy determines the amount of carotenoids that can either be used for integument colouration or for the immune system (Pérez-Rodríguez 2009). Consistently, less intense integument colouration and lower volumes of circulating carotenoids towards the city centre support the idea that the entire urban food web is deprived of carotenoids, as no association between carotenoids and ectoparasite infection intensities was found. Kestrel nestlings in more urbanised areas had a more bluish face colouration, an overall less saturated colouration and were duller, together with a lower carotenoid concentration in the bloodstream. In comparison, nestlings in less urbanised areas were more yellowish coloured—this is despite the known dietary shift that would suggest carotenoid richer prey types (like birds, shrews, lizards and insects) to be taken in urban centres and voles that are poorer in carotenoid content to be the main prey in suburban and rural areas.

Carotenoids have multiple functions in both plants (the synthesisers) and animals (consumers). Apart from being antioxidants, carotenoids play a role in UV protection, and cell membrane stability, and are used as colour pigments in skin, scales and feathers and as photosynthetic pigments in plants (Britton 2008). These functions can be affected by urbanisation and heat (Sumasgutner, Cunningham et al. 2023)—which includes the urban heat island effect whereby urban centres are usually several degrees warmer than the surrounding natural areas (Oke 1982; Imhoff, Zhang et al. 2010). In plants, heat stress, UV exposure and air pollution affect carotenoid function and level (e.g. (Camejo, Rodríguez et al. 2005; Joshi and Swami 2009)). Effects on the carotenoid synthesising trees can influence the carotenoid content in the entire food web (i.e. lower carotenoid levels in city trees (Isaksson 2009; Khosropour, Attarod et al. 2018), followed by a lower carotenoid content in urban caterpillars (Isaksson and Andersson 2007) and urban insectivores that are in turn important prey sources of urban kestrels (Sumasgutner, Krenn et al. 2013)). This together with other changes in diet quality can lead to physiological constraints that affect morphology (Olson and Owens 1998). Carotenoid-based plumage coloration is paler in urban than rural great tits *Parus major* (Isaksson, Örnborg et al. 2005), and carotenoid-based integument colouration is paler in kestrels ((Sumasgutner, Adrion et al. 2018) and this study). Our results on lower volumes of circulating carotenoids in urban centres add to these previous findings on colouration. Thus, we assume bottom-up effects, with primary producers synthesising less carotenoids—which could be associated to coping with environmental stress, including heat, in more urbanised areas—affecting all trophic levels.

Concerning ectoparasite infection intensity, no correlation with coloration or circulating carotenoids could be revealed—contrasting results of a previous investigation in the same system. Sumasgutner et al. (2018) found that while ectoparasite burden in kestrel nestlings had no direct association to the urban gradient, infection intensity was higher in nestlings with less intense face skin yellowness and was also higher in earlier broods. This inconsistency could be due to two aspects: first, in our study years, many nestlings had no ectoparasites—something we consider unusual in the study population. Thus, the result could be a year effect. Second, we adjusted the methodology from scoring parasite infection in ordinal categories (Sumasgutner, Adrion et al. 2018; Wemer, Hegemann et al. 2021) to an actual count of parasites per individual nestling to increase data resolution and variance.

Well known is an association between carotenoids and developmental stage, whereby integument colouration and blood concentrations increased both with nestlings’ age. This could be due to an improved absorption of carotenoids as well as an accumulation over time and was also reported in other raptor populations (Casagrande, Costantini et al. 2007; García-Heras, Arroyo et al. 2017). However, no association between carotenoids and body mass was seen, which could indicate that integument coloration and blood concentration depend on the quality (carotenoid content) of the ingested prey items rather than the quantity of food, as mentioned in other studies (Eeva, Sillanpää et al. 2009; Sternalski, Mougeot et al. 2010; Sternalski, Mougeot et al. 2012; Nebel, Amar et al. 2021). This could also be one reason why no relationship between carotenoids and hatch rank was found either, because hatch rank is directly informed by nestling size and was assigned in the field. Finally, we found a positive correlation between brood size and circulating carotenoids whereby nestlings in larger broods had a higher concentration of circulating carotenoids than nestlings in smaller broods. This could be directly linked to the fact that kestrel broods in suburban and rural areas are consistently larger (Sumasgutner, Nemeth et al. 2014a; Sumasgutner, Schulze et al. 2014b) than those in inner city districts, supporting the idea of larger broods in areas with higher food quality and hence carotenoid availability—which are suburban and rural areas.

## Conclusions

Our results suggest that urbanisation has an impact on prey with accumulative effects into higher trophic levels. Diet quality (carotenoid content) likely decreased with a higher degree of urbanisation, resulting in a more bluish face colouration of kestrel nestlings and especially a less saturated (less intense) integument colouration. Moreover, we found no correlation between carotenoids and ectoparasite load. Our finding supports the hypothesis that the entire urban food web is carotenoid deprived and only low-quality prey with low carotenoid content is available (e.g. fewer carotenoids in urban trees, insects, small birds, kestrels). The alternative hypothesis that nestlings allocate carotenoids as antioxidants to reduce physiological stress and/or to cope with ectoparasites rather than allocate carotenoids as pigments to coloration could therefore not be supported in this descriptive study but would require a more experimental approach. Our results might reinforce the assumption of Costantini and Møller (2008) that carotenoids only play a minor role as antioxidants in birds and that it would be important to conduct further studies considering several species at different stages within their life cycle to achieve a more complete understanding of the carotenoid pathway in avian systems.

## Figures and Tables

**Fig. 8:**
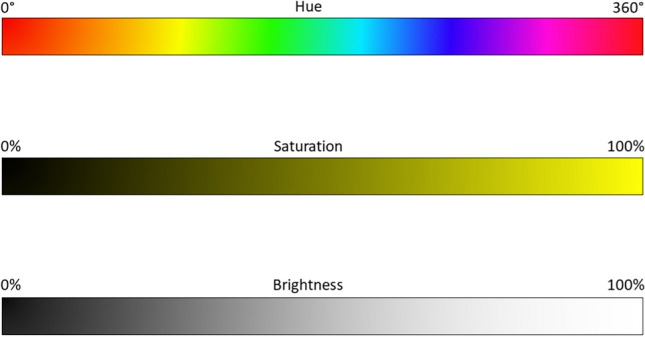
Illustration of the colour values hue (0°-360°), saturation (with yellow as an example) (0%-100%) and brightness (0%-100%)

**Fig. 9:**
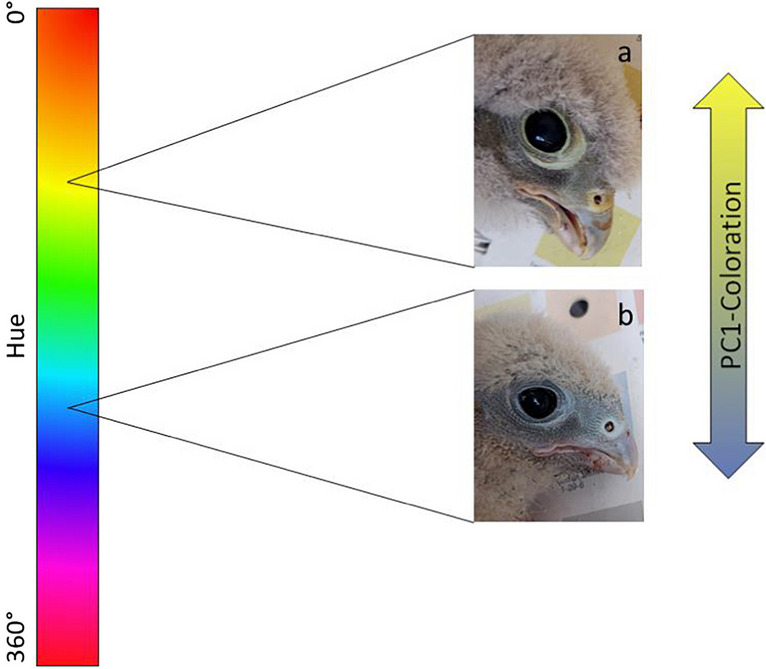
Illustration of the hue gradient with two examples of the face hue a) yellowish colouration b) bluish colouration respresented by the PC1-Colouration. High values in PC1-Colouration were represented by a yellowish colouration and low values were represented by a bluish colouration.

## Data Availability

The data underlying this study will be made available as supplementary electronic material upon acceptance of the manuscript.

## Appendix

**Table 3:** Results of the Principal Component Analysis conducted on the six-colouration measurement (i.e., hue, saturation and brightness of the face and the tarsi) of kestrel nestlings (*n*=112)

	PC1	PC2	PC3
Face Hue	**-0.982**	-0.171	0.046
Tarsi Hue	-0.003	-0.200	-0.153
Face Saturation	0.104	-0.226	0.190
Tarsi Saturation	0.157	**-0.899**	0.253
Face Brightness	-0.001	-0.241	**-0.785**
Tarsi Brightness	0.031	-0.115	**-0.508**
Importance of components:			
Standard deviation	51.281	15.127	13.65
Proportion of Variance	0.823	0.072	0.058
Cumulative proportion	0.823	0.895	0.953

Highest factor loading in each principal component are highlighted in bold
